# Brief Report: Testing the Psychometric Properties of the Spence Children’s Anxiety Scale (SCAS) and the Screen for Child Anxiety Related Emotional Disorders (SCARED) in Autism Spectrum Disorder

**DOI:** 10.1007/s10803-018-3774-8

**Published:** 2018-10-17

**Authors:** Sophie Carruthers, Rachel Kent, Matthew J. Hollocks, Emily Simonoff

**Affiliations:** 1 Institute of Psychology, Psychiatry, and Neuroscience, Department of Child & Adolescent Psychiatry, King’s College London, 16 De Crespigny Park, Denmark Hill, London, SE5 8AF UK; 2 Department of Clinical Psychology, Norwich Medical School, University of East Anglia, Norwich, NR4 7TJ UK; 3 Institute of Psychology, Psychiatry and Neuroscience, Department of Psychology, King’s College London, 16 De Crespigny Park, Denmark Hill, London, SE5 8AF UK

**Keywords:** Autism spectrum disorder, Anxiety, Psychometrics, Screening tools

## Abstract

Anxiety is a prevalent and impairing co-morbidity among individuals with autism spectrum disorder (ASD), yet assessment measures, including screening tools, are seldom validated with autism samples. We explored the psychometric properties of the child and parent reports of the Spence Children’s Anxiety Scale (SCAS) and the Screen for Anxiety Related Disorder-71 (SCARED-71) with 49 males with ASD (10–16 years, 63% co-occurring anxiety). Both measures had excellent internal consistency and fair-good parent–child agreement. The SCAS has a higher proportion of items evaluating observable behaviors. Predictive power of the measures did not differ. Higher cut-points in the parent reports (SCARED only) and lower cut-points in the child reports may enhance prediction in this sample. Choice of measure and cut-points should be considered alongside intended purpose.

Anxiety is the most common co-occurring psychiatric problem among individuals with autism spectrum disorders (ASD) (Simonoff et al. 2008), and has been associated with increased impairments in adaptive functioning and independence (White et al. 2009). Individuals with ASD and their parents/carers have reported it is a research priority for them (Wallace et al. 2013). Questionnaires are often used in both research and clinical services to screen for anxiety and to follow symptom severity over the course of intervention. Among the most frequently used are the Multidimensional Anxiety Scale for Children (MASC; March et al. 1997), Revised Children’s Anxiety and Depression Scale (R-CADS; Chorpita et al. 2000), Screen for Child Anxiety Related Emotional Disorders (SCARED; Birmaher et al. 1997) and the Spence Children’s Anxiety Scale (SCAS; Spence 1998) (Wigham and McConachie 2014). However, such questionnaires have mostly been designed for and validated with typically developing (TD) populations, which means both item content and cut-offs indicative of clinical levels of anxiety have been based on TD samples (MacNeil et al. 2009). Though research indicates some similarities in how anxiety presents in individuals with ASD relative to TD populations (in triggers, symptoms and signs of anxiety), atypical aspects of their experience and manifestation of anxiety have also been identified (Ozsivadjian et al. 2012; White et al. 2012). Of note, are associations between anxiety and sensory sensitivities (Green and Ben-Sasson 2010), disruption of routine, social confusion (Ozsivadjian et al. 2012) and unusual fears (Mayes et al. 2013). Furthermore, research suggests that anxiety among the ASD population is more commonly expressed through externalizing behaviors than verbalization (Evans et al. 2005). In addition, the difficulties experienced by people with autism in recognizing and communicating their emotions may influence how questionnaires are understood and completed. Hence, items about internal mental states may be less reliably endorsed. In line with these differences, recent studies have demonstrated that questionnaire factor structure is different in those with ASD compared to TD individuals with anxiety disorders and healthy controls (Glod et al. 2017; Magiati et al. 2017; White et al. 2015). Many different measures are currently used and there is no consensus as to which are most useful in ASD populations (Wigham and McConachie 2014). A better understanding of how different anxiety instruments may perform with an ASD population is needed.

We were interested in comparing the performance of parent and child reports of two commonly used measures as screening instruments within the same sample. The Spence Children’s Anxiety Scale (SCAS; Spence 1997) and the Screen for Child Anxiety Related Emotional Disorders in Children-71-item version (SCARED-71; Bodden et al. 2009) are available for both parent and child report and have good psychometric properties in TD populations (Bodden et al. 2009; Spence et al. 2003). A previous Dutch study using the SCARED-71 with a clinically anxious ASD sample of children demonstrated both child and parent versions (hereafter referred to as SCARED-C and SCARED-P, respectively) have good internal consistency and construct validity (van Steensel et al. 2013). Though for each subscale sensitivity was good, specificity was low and raising cut-offs for the separation, social and generalized anxiety subscales above those recommended for TD populations increased the subscale specificity rates and overall accuracy of identification. In contrast, change in subscale cut-off for the child report did not result in improved identification of anxiety (van Steensel et al. 2013). The authors could not examine the appropriateness of total score cut-offs as their entire sample had anxiety diagnoses, and this has yet to be explored.

Among populations of children with ASD, the SCAS child (SCAS-C) and parent (SCAS-P) have demonstrated good–excellent internal consistency (Magiati et al. 2014; Zainal et al. 2014), with the SCAS-P reported to have good convergent (Zainal et al. 2014; Magiati et al. 2017), divergent and discriminant validity (Magiati et al. 2017). There is limited use among previous studies of diagnostic tools to prospectively identify anxiety diagnoses. Using a cut-off of one standard deviation above the normative mean, a sensitivity of .71 and specificity of .76 has been reported for the SCAS-P when identifying children with ASD with at least one key symptom of an anxiety disorder on the Kiddies-SADS (Zainal et al. 2014). Such analysis has not been conducted with the SCAS-C, nor has the relative performance of alternative SCAS cut-offs than those published for TD populations been explored. Moreover, no study has yet directly compared the performance of both measures within the same sample.

Parent–child agreement correlations on individual measures are expected to be around 0.3–0.4 among the general population (Achenbach et al. 1987). In ASD populations, agreement on the SCAS ranges between 0.25 and 0.69 (Magiati et al. 2014; May et al. 2015; Ozsivadjian et al. 2014; Ooi et al. 2016), while no such reports are available for the SCARED-71. Ooi et al. 2016 suggest that parent–child agreement in SCAS may be higher on items describing observable behaviors.

Our study aimed to examine the psychometric properties and relative performance of the SCARED-71 and SCAS anxiety questionnaires in a sample of adolescent boys with ASD. With the use of a structured diagnostic interview to prospectively identify diagnoses and parent and child questionnaire reports, we wished to compare the characteristics of the two questionnaires and to explore whether there were differences in their parent–child agreement, reliability and predictive validity that would suggest one may provide a better screening tool for anxiety in youth with ASD.

## Method

### Participants

Participants were a subset of individuals from a sample of 52 adolescent boys who participated in a study exploring the cognitive and physiological correlates of anxiety in youth with ASD (Hollocks et al. 2014, 2016). Data from this sample were also included in a pooled sample exploring psychometric properties of the SCAS-P (Magiati et al. 2017). All analyses in the present study were limited to the 49 participants with complete data for both parent- and child-report anxiety questionnaires and parent diagnostic interview. Boys aged 10–16 years (M = 12.88, SD = 1.92) with a clinical diagnosis of ASD were recruited primarily from National Health Service (NHS) clinics in London and the southeast of England. ASD diagnoses were confirmed by local clinicians, and additional confirmation of the diagnoses was carried out for 27 of the 49 participants using the Autism Diagnostic Interview-Revised (ADI-R; Lord et al. 1994) and/or the Autism Diagnostic Observation Schedule (ADOS; Lord et al. 2000). In the absence of ADOS/ADI confirmed diagnosis, a parent-reported Social Communication Questionnaire—lifetime version (SCQ; Rutter et al. 2003) score of ≥ 15 in combination with a clinical diagnosis was required. All participants had an IQ ≥ 70. Participants were excluded if they were currently taking any medications for anxiety or depression. The same parent completed all parent-report measures. The study was approved by the South East London Research Ethics Committee (REC 4: 10/H0870/67).

### Measures

The Screen for Child Anxiety Related Disorder-71 (SCARED-71; Bodden et al. 2009) is a 71-item questionnaire with separate versions for parents (SCARED-P) and children (SCARED-C). There are nine subscales: panic disorder, generalized anxiety disorder, separation anxiety, social phobia, obsessive–compulsive disorder, post-traumatic stress disorder, animal phobia, blood-injection-injury phobia, and situational-environmental phobia. In a TD population, Cronbach’s alpha for the total scores was measured at 0.95 for the child version and 0.96 for the parent version (Bodden et al. 2009). Total cut-off scores of 30 and 21 have been identified for the child- and parent-report version, respectively; the child version has a reported sensitivity of 0.78 and a specificity of 0.76, and for the parent version the sensitivity was 0.92 and specificity 0.92.

The Spence Children’s Anxiety Scale (parent and child versions, SCAS-P and SCAS-C; Spence 1998) is a questionnaire with 38 scored items. There are six subscales: separation anxiety, social phobia, obsessive compulsive disorder, panic/agoraphobia, physical injury fears, and generalized anxiety. Among TD populations, the SCAS has a good internal consistency (*α* = .60–.92) and moderate 6-month test–retest correlation coefficient of .62 (Spence 1998). T-scores and cut-offs for the SCAS-C and SCAS-P are available on the SCAS website according to age and gender. With the SCAS-C, for boys aged 12–15 years, a total score cut-off of ≥ 33 is suggested. There are no available suggested cut-offs for children aged 16 years so the threshold of ≥ 33 was used for boys of this age (n = 4). For boys aged 8–11 years, a total score of ≥ 40 is suggested. However, this current sample has too few cases in the younger age group to stratify cut-point analyses by age and therefore 33 was used for all participants. With the SCAS-P, cut-offs are only available for children up to age 13 years. For boys aged 10–13 years, a total score cut-off of ≥ 24 is suggested. In a study of TD participants (aged 6–18 years), using the cut-off of 24 (which at that time was only a recommended ‘clinical caseness’), the SCAS-P has correctly classified 86% of the anxiety disordered and 71% of the non-anxiety controls (Nauta et al. 2004). In the absence of official threshold recommendations for boys older than 13 years, the cut-off of 24 will be used for all participants on the SCAS-P.

Both scales include items that investigate both observable behaviors (e.g. my child complains of feeling afraid) and internal states (e.g. my child worries what other people think of him/her). Two of the authors (SC & RK) independently rated all parent-report items as ‘observable’ or ‘not observable’. Observable behaviors were considered those that someone other than the child (e.g. parents or carers) could have reasonable observable evidence for, rather than being based on an assumption of what their child was thinking or feeling.

The Child and Adolescent Psychiatric Assessment (CAPA; Angold et al. 1995) is a semi-structured psychiatric interview schedule eliciting DSM-IV diagnoses present in the last 3 months. The CAPA has previously been successfully used in samples with ASD (Simonoff et al. 2008). As in previous studies, parent report was used in the current study and is described in more detail elsewhere (Hollocks et al. 2014). Diagnoses of interest were separation anxiety, generalized anxiety disorder (GAD), specific phobia, social phobia, agoraphobia and panic disorder. OCD and PTSD were allowed to be co-occurring anxiety disorders alongside additional anxiety diagnoses but not the sole diagnosis. Social Communication Questionnaire—lifetime version (SCQ; Rutter et al. 2003) was used as an index of autism symptom severity.

Strengths and Difficulties Questionnaire (SDQ; Goodman 1997) measures emotional and behavioral symptoms. The SDQ comprises five subscales: hyperactivity, conduct problems, emotional symptoms, peer relationship problems, and prosocial behavior. To provide a measure of behavioral difficulties, which are frequently seen in autistic adolescents with and without co-occurring anxiety disorders, we used the hyperactivity and conduct problems subscales. We did not include the emotional subscale as it shares too much overlap with anxiety. Wechsler Abbreviated Scale of Intelligence (Wechsler 1999) was used to measure full-scale IQ.

### Statistical Analyses

Data analysis was conducted using Stata 14. Alongside recognized descriptive psychometric statistics, the level of parent–child agreement for the SCAS versus the SCARED was compared using a structural equation model with a maximum likelihood estimator. In this model, SCARED-P, SCARED-C, SCAS-P and SCAS-C total scores were observed variables and two latent variables representing “shared variance” were estimated, one for each questionnaire, respectively. To determine whether these shared variances differed significantly, the model was then constrained to estimate a single latent variable for the shared variance of the two questionnaires overall (Griffin and Gonzalez 2012). A Chi square test was used to test the difference between the two ICC estimators using the *testnl* command. Predictive validity of the measures and relative cut-offs were assessed using receiver operating characteristic (ROC) implemented with the procedure *roctab* through which sensitivity, specificity, positive predictive values (PPV), negative predictive values (NPV), area under the curve (AUC) values and alternative cut-offs were examined. We determined the sensitivity and specificity for the published cut-offs and also identified cut-offs at which the percentage correctly classified was greatest. Where this was achieved at multiple cut points, the one associated with the highest sensitivity is given in Table 2 (as screening instruments are typically used to ensure cases are not missed). The predictive validity of the two measures was compared using *roccomp*. Hierarchical regressions were used to explore if adding parent-report to child-report improved prediction, and vice versa.

## Results

### Anxiety Diagnoses

Using the DSM-IV CAPA algorithms, 31 participants (63%) met criteria for one or more anxiety diagnosis: 25% met criteria for separation anxiety; 39% for GAD; 4% for specific phobia; 8% for social phobia; 16% for agoraphobia, 27% for panic disorder and 18% for OCD. No individuals received a PTSD diagnosis. Eight individuals (16%) met criteria for one diagnosis, 13 (27%) for two diagnoses, 10 (20%) individuals for three or more diagnoses. Of those who met criteria for OCD, only 1 had this as their only anxiety diagnosis. This individual was excluded from further analysis.

### SCAS and SCARED-71

Participants with anxiety diagnoses scored significantly higher than those without anxiety diagnoses on all anxiety questionnaires (Table 1).

**Table 1:** Sample characteristics, test scores and t-test results for each anxiety questionnaire for individuals with and without an anxiety diagnosis

Measure	Total sample (n = 48)	With anxiety diagnosis (n = 30)		Without anxiety diagnosis (n = 18)	
	Mean score (SD)	Mean score (SD)	Range	Mean score (SD)	Range
SCARED-child (C)*	46.1 (22.7)	51.4 (23.5)	7–108	37.3 (18.7)	9–70
SCARED-parent (P)**	53.8 (23.5)	61.9 (23.2)	17–108	40.2 (17.4)	12–74
SCAS-child (C)**	31.1 (16.9)	35.9 (17.8)	5–72	23.1 (11.7)	3–38
SCAS-parent (P)***	33.8 (18.7)	41.6 (18.2)	11–88	20.8 (10.7)	6–45
Age	12.9 (1.9)				
IQ	101.3 (13.0)				
SCQ	22.8 (6.5)				
SDQ	22.2 (6.7)				

*SCQ* Social Communication Questionnaire, *SDQ* Strengths and Difficulties Questionnaire, IQ as measured by the Wechsler Abbreviated Scale of Intelligence (Wechsler 1999)

*p < 0.05; **p < 0.01; ***p < 0.001, independent samples t-test between those with and without an anxiety diagnosis

### Item Content: Observability

For the SCARED-P, the raters agreed on 59 of 71 items (83.1%). Of the agreed items, 24 (40.7% of the agreed items) were considered to rate observable behavior. For the SCAS-P, the raters agreed on 32 of 38 items (84.2%). Of those items that were agreed upon, 21 (65.6%) items were deemed observable.

### Internal Consistency

There was excellent internal consistency for both the SCARED-71 (Child: Cronbach’s *α* = 0.95; Parent: *α* = 0.95) and the SCAS (Child: *α* = 0.93; Parent: *α* = 0.94). Cronbach’s alpha values for the subscales ranged from 0.65 to 0.88 for the SCARED-C, 0.57–0.90 for the SCARED-P, 0.72–0.82 for SCAS-C and 0.62–0.83 for the SCAS-P. Subscale alphas were above the 0.70 level considered as acceptable except for the SCARED-OCD for parents, SCARED-PTSD for both children and parents, SCARED-Situation phobia for both children and parents and the SCAS-Physical injury for parents.

### Correlations Across Questionnaires and Parent–Child Agreement

Across measures and within raters, the child (Pearson *r* = 0.85, p < 0.001) and parent (*r* = 0.94, p < 0.001) versions were highly correlated. Across raters and within measures (parent–child agreement), the ICC for the SCAS was .59 (95% confidence intervals (CI) .41–.77), which was significantly higher than the ICC for the SCARED-71 of .38 (95% CI .21–.54), Χ^2^_1_ = 44.42, p < 0.001.

### Predictive Validity

AUC values (Table 2) demonstrate moderate-good predictive validity across the measures. The relative sensitivity and specificity values are compared for the original and optimal cut-offs in Table 2. ROC comparisons did not find any significant differences in the AUC between the two parent measures, the two child versions or the parent and child versions of each measure (p > 0.05). Inspection of alternative cut-points revealed very similar rates of correct classification with varying scores for the SCARED-P, all of which were higher than the original cut-off. For the child measures, these led to considerably lower cut-points (SCARED: 21 vs. the original 30, SCAS-C: 13 vs. 33) while for the parent this was not consistent (SCARED: 38 vs. the original 21, SCAS: 24 which was the same as the original). We observed a few older children (13–15 years) who had received an anxiety diagnosis who scored very low on the child self-report measures.

**Table 2:** AUC, sensitivity, specificity, positive predictive value (PPV) and negative predictive value (NPV) of the measures using recommended cut-offs for TD samples and alternative cut-offs for this ASD sample using receiver operating curve (ROC) analyses

Measure	AUC (95% CI)	Cut-off	Correctly classified (%)	Sensitivity	Specificity	PPV	NPV
SCARED-C—*original*	0.67 (0.51–0.83)	30	66.7	0.83	0.39	0.69	0.58
SCARED-C—*alternative*		21	70.8	0.93	0.33	0.70	0.75
SCARED-P—*original*	0.77 (0.64–0.91)	21	66.7	0.97	0.17	0.66	0.75
SCARED-P—*alternative*		38	72.9	0.87	0.50	0.74	0.69
SCAS-C—*original*	0.73 (0.59–0.87)	33 (40)^a^	56.7	0.57	0.78	0.81	0.52
SCAS-C—*alternative*		13	68.8	0.90	0.33	0.69	0.67
SCAS-P—*original*	0.84 (0.73–0.95)	24	81.3	0.90	0.67	0.82	0.80
SCAS-P—*alternative*		24	81.3	0.90	0.67	0.82	0.80

*AUC* area under the curve, *CI* confidence interval, SE standard error; *PPV* positive predictive value, *NPV* negative predictive value

^a^SCAS-C cut-off of 33 has been used for whole sample but there is a recommended cut-off of 40 for children aged 8–11 years

### The role of multiple measures in predicting diagnosis

Using logistic regressions, we examined whether the predictive relationship between each anxiety questionnaire and the presence of an anxiety disorder was modified by the inclusion of additional characteristics: autism symptoms (SCQ), behavioral difficulties (combined hyperactivity and conduct subscales on the SDQ) and IQ (WASI). For both measures and across parent and child reports, the findings were the same: child’s IQ and the presence of behavioral difficulties did not modify the relationship to anxiety diagnosis but in all cases a higher score on the SCQ also significantly predicted greater likelihood of an anxiety diagnosis (p’s ranging from 0.010 to 0.038). However, in all cases, the SCAS or SCARED remained a significant predictor when autism severity was included, indicating that these measures are robust to different levels of autism severity. In order to determine whether information from both parent and child improved diagnostic accuracy, within measure we added the child report to the logistic regression of parent report on anxiety diagnosis. In neither case did the addition of child-report improve overall prediction (ΔR^2^ p = 0.445, 0.710 for SCARED and SCAS respectively). For the converse, however; adding parent to child report led to improvement in model fit for both the SCARED-71 (p = 0.012) and for the SCAS (p = 0.003).

## Discussion

Our study is the first to compare both parent- and child-rated versions of two of the most commonly used anxiety questionnaires, originally validated for TD populations, with an ASD sample. Our results indicate that those who received at least one anxiety diagnosis scored significantly higher on both parent and child report of both questionnaires. A comparison of the two measures’ parent and child reports revealed no difference in their ability to predict anxiety diagnoses. Both questionnaires had excellent internal consistency for total scores across both reports with most subscales reaching acceptable levels. Across measures, there were high correlations between the relative parent and child report suggesting consistency across the different tools.

The levels of agreement between parents and children are similar, if slightly higher, to those reported for TD samples (Achenbach et al. 1987), and higher for the SCAS than the SCARED-71. A possible reason is the higher proportion of items in the SCAS-P that were identified to be based on observable behavior. Previous research suggests behaviors that are directly observable by parents have higher parent–child agreement (Ooi et al. 2016; Blakeley-Smith et al. 2012). This may be particularly desirable in ASD, where young people are more likely to have difficulties in verbalizing their internal experiences. Overall, the ROC comparison revealed no difference in the predictive validity of the two measures, suggesting neither is a particularly stronger measure in this sample than the other. In interpreting this lack of discrimination, however, the relatively small sample size and hence the low power should be borne in mind. It is worth reflecting that despite the SCAS being significantly shorter in length, it performed just as well as the SCARED-71, which may appeal to researchers and clinicians when length of measures is a consideration.

Our data suggest alternative cut-off scores may increase the proportion of correctly classified individuals; in particular, lower cut-offs performed better for the child reports. This highlights the importance of not using currently published cut-offs for clinical purposes in youth with ASD. Larger and more varied populations are required to better understand the optimal indicative thresholds for both measures in people with autism. In a few cases we observed very low scores on the self-report measures among some of the older children with diagnoses. This suggests that very low scores in adolescents and older children should be interpreted with caution when there is other evidence that anxiety is present as this may reflect emotional literacy issues.

Both measures had moderate-good predictive validity. Parent-report measures showed slightly higher predictive validity than the child self-report measures, which was consistent with the finding that adding child report to parent report did not improve prediction of anxiety diagnosis for either questionnaire, but that adding parent report to child report improved prediction for both questionnaires. However, anxiety diagnoses were based on a parent-reported psychiatric interview which limits the conclusions we can draw from this particular finding. We prioritized parent reports because a number of the adolescents had difficulties with the open structure of the CAPA questions and reporting of internal mental states. This would not be expected in a TD sample of this age and verbal ability. In our sample, level of autism severity but not IQ or behavioral difficulties was also associated with an anxiety diagnosis and future studies should consider whether this modifies the accuracy or cut-points for different screening measures.

These results should be interpreted in the context of a number of limitations, including a relatively small sample size, high ability ASD group, and insufficient numbers of cases in the younger children to stratify cut-point analysis by age. With regards to parent–child agreement, as in many studies, we took no specific steps to prevent the parent and child completing the measures together. We also did not explore the possible influence of parental education or socioeconomic status on levels of parent–child agreement. Diagnostic criteria changes between DSM-IV and DSM-V are minimal in relation to the diagnoses reported and should not limit generalization of findings. Future studies are needed to explore validation of anxiety measures in ASD samples with larger ranges of IQ, females and older adolescents and adults.

Though these findings add to the existing literature suggesting these two commonly used questionnaires are reasonably valid tools for use with ASD populations, neither measure will capture the more nuanced aspects of anxiety that seem unique to ASD (Kerns and Kendall 2012; Ozsivadjian et al. 2012; Trembath et al. 2012; White et al. 2012). There is therefore a risk of underestimating the true severity of anxiety among ASD populations with such measures and it is important to continue to develop specialist tools for measuring anxiety in ASD (Rodgers et al. 2016; Kerns et al. 2017).

In conclusion, both the SCAS and SCARED are suitable for use with an ASD population, demonstrating acceptable levels of predictive validity, although the cut-offs are not valid. Similar evaluations in broader samples are needed to better understand the value of screening tools in this population. Our work supports the ongoing effort to develop specific tools for measuring anxiety in ASD populations.
